# Comparative Genomics Reveals Unique Genetic Determinants of Biofilm Formation in *Campylobacter*

**DOI:** 10.3390/ijms27062543

**Published:** 2026-03-10

**Authors:** Yiping He, Gretchen Dykes, Heather Koppenhöfer, Joseph Capobianco, Chin-Yi Chen

**Affiliations:** Characterization and Interventions for Foodborne Pathogens Research Unit, Eastern Regional Research Center, Agricultural Research Service, United States Department of Agriculture, 600 East Mermaid Lane, Wyndmoor, PA 19038, USAchin-yi.chen@usda.gov (C.-Y.C.)

**Keywords:** *Campylobacter*, biofilm, whole-genome sequencing, comparative genomics, pangenome analysis, foodborne pathogen, food safety

## Abstract

A biofilm is a complex microbial community that protects bacterial cells from various stressors, including harsh environmental conditions, antimicrobial treatments, and host immune responses. This protective capability enhances *Campylobacter* survival during food processing and storage and facilitates transmission to humans. Despite their importance, the molecular mechanisms underlying *Campylobacter* biofilm formation and its impact on pathogen persistence remain poorly understood. In this study, we characterized the biofilm-forming ability of 18 *C. jejuni* and *C. coli* strains isolated from retail meat and performed whole-genome sequencing and comparative genomic analysis to identify strain-specific genes contributing to biofilm formation and maintenance. Phenotypic analysis revealed that *C. jejuni* strains YH001 and YH027 exhibited the strongest biofilm-forming capacity, producing the highest biomass among all isolates. Phylogenetic analysis indicated a close genetic relationship between these two strains, while pangenome analysis identified 19 unique genes/proteins specific to these strains. Functional annotation indicated their critical roles in adhesion, extracellular matrix production, and stress response. These findings demonstrate strain-specific biofilm formation in *Campylobacter* and highlight genetic determinants that may serve as targets for novel therapeutic approaches and intervention strategies to disrupt biofilms, improve food safety, and reduce persistent infections.

## 1. Introduction

*Campylobacter* is one of the most common foodborne pathogens worldwide and a leading cause of human gastroenteritis, with typical symptoms including diarrhea, abdominal pain, fever, and nausea. According to the Centers for Disease Control and Prevention (CDC), approximately 1.5 million cases of *Campylobacter* infection occur each year in the United States, making it a major public health concern [1]. Among the various species, *Campylobacter jejuni* and *C. coli* are most commonly associated with human illness [2]. These species are prevalent in poultry and other animal-derived food and can also be found in eggs, unpasteurized milk, and untreated water. The primary routes of transmission to humans include the consumption of undercooked poultry, cross-contamination in kitchens, and the ingestion of contaminated water or raw milk [3,4].

Biofilms are structured microbial communities in which cells are aggregated and embedded in a matrix of extracellular polymeric substances (EPS). This matrix, produced either by the cells themselves or by surrounding bacteria, consists of polysaccharides, structural proteins (e.g., flagella and pili), nucleic acids (extracellular DNA (eDNA) and RNA (eRNA)), lipids, and other biomolecules [5]. Polysaccharides are essential for biofilm formation contributing to cell adhesion to surfaces and the maintenance of structural integrity [6]. Biofilm proteins play important roles in biofilm development and the survival of cells by facilitating access to nutrients and regulating biofilm integrity. They are involved in attaching cells to surfaces, developing three-dimensional biofilm structures, and maintaining biofilm stability through interactions with exopolysaccharide and nucleic acids [7,8]. As a key component of biofilm structure, eDNA contributes to biofilm formation, adhesion, and structural integrity by binding and connecting cells within biofilms [9].

Biofilms formed by pathogenic bacteria present a major challenge in the food processing industry. Common foodborne pathogens, including *Campylobacter*, *Salmonella*, *Escherichia coli* O157:H7, *Listeria monocytogenes*, and *Staphylococcus aureus*, readily develop biofilms on both biotic and abiotic surfaces during food processing and storage. These biofilms significantly enhance bacterial resistance to environmental stresses, disinfectants, and antibiotics, making intervention and eradication efforts difficult [10]. It has been reported that biofilm-embedded cells can exhibit remarkably higher (100–1000-fold) resistance to antimicrobials compared with planktonic cells due to the protective properties of the matrix and altered physiology [11]. Consequently, biofilms contribute to persistent contamination, reduced sanitation efficacy, and elevated food safety risks across production environments.

*Campylobacter* is a Gram-negative, spiral-shaped, microaerophilic bacterium that thrives in low-oxygen environments, typically requiring 3–10% oxygen, 5–10% CO_2_ and a temperature of 37–42 °C for optimal growth. These physiological constraints favor its persistence in niches such as the gastrointestinal tract of animals, as well as in water systems and plumbing within animal husbandry facilities and food processing plants. In these environments, *Campylobacter* frequently forms biofilms. Studies have shown that *C. jejuni* cells embedded within a biofilm matrix exhibit significantly greater tolerance to environmental stresses, such as oxygen exposure and temperature fluctuations, compared to planktonic cells [12]. This enhanced resilience makes biofilm-associated *Campylobacter* extremely difficult to eradicate, leading to persistent contamination in food processing facilities and increasing the risk of foodborne infections [12,13,14,15].

*Campylobacter* biofilm formation exhibits significant strain-to-strain variation influenced by genetic factors and environmental conditions including nutrient limitation, extracellular DNA, oxygen availability, and interactions with co-cultivated bacteria [16,17]. Despite its importance for persistence and transmission, the molecular basis of biofilm development in *Campylobacter* remains poorly understood. Although previous studies have suggested individual genes linked to flagellar synthesis, stress response, and quorum sensing that influence biofilm formation, the broader genetic determinants and regulatory networks have yet to be systematically characterized [18,19,20].

The rapid expansion of whole-genome sequencing has greatly enhanced our understanding of genetic diversity within *Campylobacter*. However, genomic data have not been fully integrated into a mechanistic understanding of biofilm formation [18]. There is currently no comprehensive validated genetic framework that explains how diverse *Campylobacter* strains coordinate biofilm development across environmental settings. This knowledge gap limits our ability to predict biofilm phenotypes from genomic data and hinders the development of targeted interventions.

To address these limitations, we conducted a combined genomic and phenotypic analysis of biofilm formation across 18 *Campylobacter* isolates (nine *C. jejuni* and nine *C. coli*) recovered from retail meat products. We assessed biofilm-forming ability, performed whole-genome sequencing, and generated phylogenetic and pangenomic profiles to identify genes and proteins associated with biofilm phenotypes. The functional annotation of genes uniquely present in biofilm-forming strains provided insights into potential mechanisms underlying biofilm development and maintenance. Together, these findings expand the current understanding of the genetic basis of *Campylobacter* biofilms and support the development of targeted strategies to enhance food safety.

## 2. Results and Discussion

### 2.1. Determination of Biofilm-Forming Ability of C. jejuni and C. coli Food Isolates

Biofilm formation was examined in 18 food-derived *Campylobacter* isolates (nine *C. jejuni* and nine *C. coli*). These isolates represent the complete set of strains recovered from retail meat samples during our surveillance effort for which high-quality complete genome sequences were generated. Thus, the isolates constitute the entire dataset available for this comparative genomic analysis, rather than a subset selected based on specific phenotypic or genotypic traits. After incubating duplicate samples in polystyrene tubes under microaerobic conditions at 42 °C for five days, adherent cells were washed and stained with crystal violet solution.

**Figure 1:**
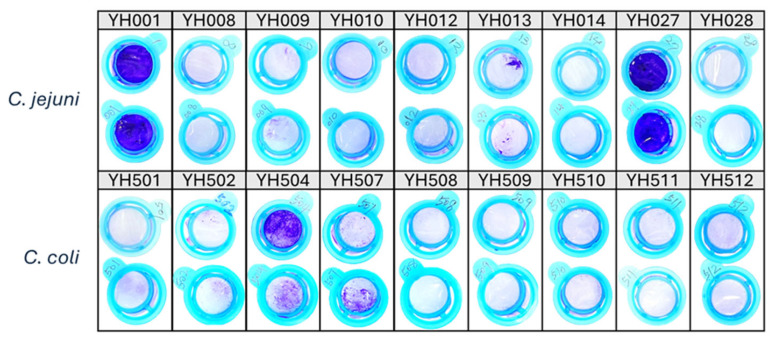
Biofilms of *C. jejuni* (upper panel) and *C. coli* (low panel) strains stained with crystal violet. Biofilms were developed in duplicate samples under microaerobic conditions at 42 °C for five days and visualized after staining.

The results are shown in Figure 1. Biofilm-forming ability varied significantly among the strains: *C. jejuni* YH001 and YH027 produced the largest amount of biofilm biomass, as indicated by crystal violet staining, whereas *C. coli* YH504 and YH507 formed moderate biofilms. The remaining strains showed negligible biofilm formation under the same conditions. The stronger adhesion and aggregation observed in *C. jejuni* YH001 and YH027 compared to other isolates indicate that biofilm formation in *Campylobacter* is strain-dependent, a finding consistent with prior studies showing variability across strains and species [21].

### 2.2. Comparison Between Genetic Relatedness and Biofilm Formation of Campylobacter Isolates

**Figure 2:**
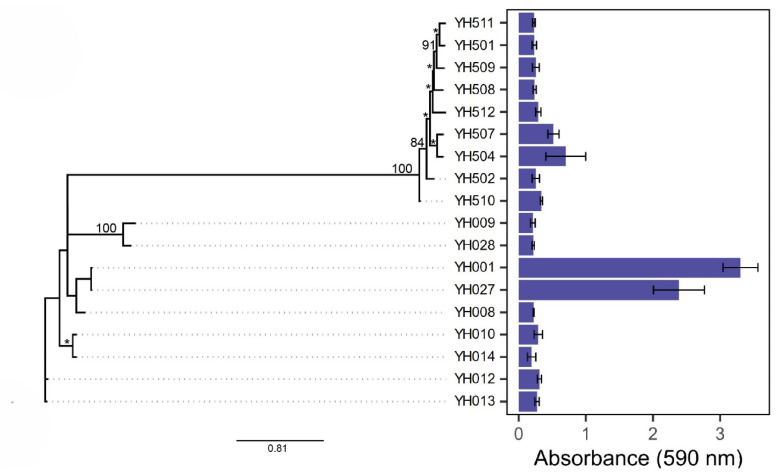
SNP-based phylogenetic tree and biofilm quantification of *C. jejuni* and *C. coli* isolates. Left panel: SNP-based phylogenetic tree constructed from whole-genome sequences of *C. jejuni* (YH001–YH028) and *C. coli* (YH501–YH512) isolates. Bootstrap values on branches correspond with maximum likelihood consensus tree. * indicates a bootstrap value of 100. Right panel: Biofilm formation quantified by crystal violet staining after 5 days of growth in Mueller–Hinton broth under microaerobic conditions at 42 °C without shaking. Absorbance at 590 nm represents the average of two independent growing biofilm samples, each assayed in duplicate. Error bars represent the standard deviation, with significance defined as *p* < 0.05.

After crystal violet staining, the biofilm biomass was dislodged and quantified by measuring absorbance at 590 nm. The results confirmed that *C. jejuni* YH001 and YH027 were the strongest biofilm-forming strains, whereas *C. coli* YH504 and YH507 formed moderate biofilms among 18 *Campylobacter* food isolates (Figure 2, right panel).

Biofilm formation in bacteria can be influenced by genetic background, environmental adaptation, and regulatory mechanisms. To compare the genotypic traits associated with *Campylobacter* biofilm formation, a phylogenetic tree was constructed based on SNPs derived from whole-genome sequences of *C. jejuni* and *C. coli* isolates (Figure 2, left panel). Interestingly, *C. jejuni* YH001 and YH027 (the two strongest biofilm producers) were clustered together in the tree, indicating close genetic relatedness and shared genomic elements. Similarly, *C. coli* YH504 and YH507 (two moderate biofilm producers) were also clustered closely in the tree. These findings suggest that biofilm-forming ability may correlate with genetic similarity, supporting the observed strain-dependent variability in *Campylobacter* biofilm formation.

### 2.3. Genetic Traits Associated with Biofilm Formation and Stability

**Figure 3:**
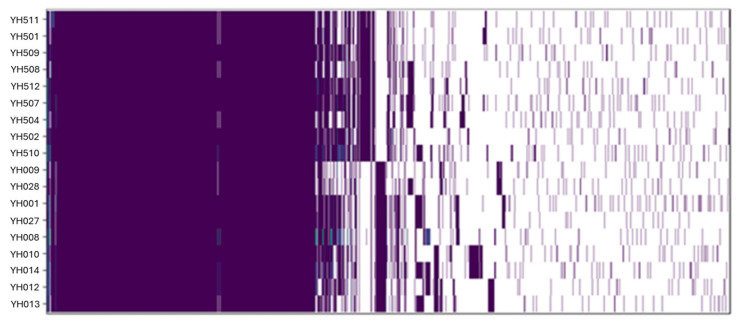
Pangenome analysis of *C. jejuni* and *C. coli* isolates. The heatmap shows the presence (purple) and absence (white) of genes or gene clusters across *C. jejuni* and *C. coli* genomes. The x-axis represents gene clusters, but their position does not correspond to chromosomal location. This visualization highlights the genetic diversity among isolates.

To identify core genes shared by all 18 *Campylobacter* isolates and variable genes unique to specific strains, a pangenome analysis was performed. The complete results are provided in Supplementary Table S1. The heatmap in Figure 3 illustrates the presence and absence of genes across *C. jejuni* and *C. coli* isolates, revealing highly diverse genetic profiles with no two strains exhibiting identical genome patterns.

### 2.4. Identification of Strain-Specific Genes Associated with Biofilm Formation

To identify genes potentially involved in *Campylobacter* biofilm formation, we searched for strain-specific genes and annotated proteins using the complete pangenome dataset (Supplementary Table S1). This analysis revealed 19 unique genes/proteins, including five hypothetical proteins, which were exclusively present in *C. jejuni* YH001 and YH027, and two strains characterized as strong biofilm producers (Table 1).

Additionally, two annotated genes (methyl-accepting chemotaxis signal transduction protein and cytolethal distending toxin subunit A) and five hypothetical genes were uniquely identified in *C. coli* YH504 and YH507, which are both moderate biofilm producers. None of these genes overlapped with those found in the strong biofilm-forming *C. jejuni* strains, suggesting that the moderate biofilm phenotype in *C. coli* may not be driven by gene content directly analogous to that associated with strong biofilm formation. Although *C. jejuni* and *C. coli* share substantial genomic homology, they remain clearly distinct species, exhibiting roughly 70–80% overlap in their core genomes and 75–85% average nucleotide identity (ANI). The observed differences in proteins associated with biofilm formation may reflect species-specific regulatory mechanisms, variations in gene expression, or functional divergence within biofilm-related pathways. The presence of multiple hypothetical genes further indicates that uncharacterized functions may contribute to biofilm formation, underscoring the need for additional functional studies to elucidate their roles.

Functional annotation suggested that the candidate genes associated with the biofilm-forming phenotype may contribute to biofilm development and stability by promoting cell adhesion, extracellular matrix production, and key regulatory signaling. In addition, we categorized the functional subsystems for the 19 unique genes associated with the observed biofilm phenotypes. Genes unique to the strong biofilm-forming strains were categorized into functional subsystems associated with virulence, disease and defense; protein, nitrogen and potassium metabolism; membrane transport; and amino acids and derivatives (Table 1).

Specifically, Dihydrolipoamide dehydrogenase (DLDH) is a central metabolic enzyme that converts pyruvate to acetyl-CoA, supporting energy production essential for bacterial survival under nutrient-limited conditions. Beyond metabolism, DLDH has been implicated in bacterial adherence, biofilm formation, structure integrity, and virulence, and has also been detected in the exopolysaccharide (EPS) matrix of *Pseudomonas aeruginosa* biofilms [22].

FAD-dependent NAD(P)-disulfide oxidoreductases catalyze disulfide bond formation, which stabilizes biofilm matrix proteins and enhances cohesion and resilience within the biofilm. These enzymes also participate in polysaccharide biosynthesis, contributing to the extracellular polymeric substance (EPS) that forms the structural backbone of the biofilm matrix [23,24].

DD-carboxypeptidase, a key enzyme in peptidoglycan (PG) biosynthesis, is essential for maintaining cell wall structure, surface attachment, and overall biofilm stability. Defects in PG synthesis impair cell–cell interactions and biofilm formation [25,26]. Studies in *C. jejuni* demonstrated that DD-carboxypeptidase mutations lead to defective PG assembly and diminished biofilm development [27].

DNA-binding protein Roi is important in structuring the biofilm matrix, which is formed largely by extracellular DNA (eDNA) together with exopolysaccharides and other components. These DNA-binding proteins act as scaffolds that cross-link eDNA to other matrix components, thereby supporting biofilm formation and maintaining matrix integrity [28].

NiFe hydrogenases, frequently detected in diverse biofilm communities, participate in hydrogen metabolism and energy conversion pathways that support microbial survival within biofilm environments [29].

L-Proline/Glycine betaine transporter (ProP) facilitates the uptake of compatible solutes necessary for osmotic balance, surface attachment, and EPS synthesis, all of which are important for biofilm formation [30].

YraQ family membrane proteins promote cell adhesion and contribute to EPS matrix production, playing a role during the early stages of biofilm establishment and structural stabilization [31].

Alpha-ketoglutarate permease (KgtP), which transports α-ketoglutarate for central carbon and nitrogen metabolism, may enhance bacterial fitness under nutrient-limited conditions typical of biofilms, thereby influencing biofilm growth and architecture [32].

Efflux proteins also play multifaceted roles in biofilm biology by exporting EPS components, quorum-sensing molecules, and other factors involved in the adhesion, aggregation, and transcriptional regulation of biofilm formation [33,34].

Ammonium transporters facilitate ammonium uptake and waste removal, both of which are critical for biofilm physiology and can influence biofilm structure and microbial survival [35].

Sodium-dependent phosphate transporters, including the PstS subunit, support phosphate acquisition and have been implicated in shaping biofilm structure and promoting biofilm formation [36].

Multi-antimicrobial extrusion proteins (MATEs) further enhance biofilm resilience by exporting antimicrobial compounds, contributing to the high tolerance commonly observed in biofilm-embedded cells [33,37].

The potassium-transporting ATPase A chain maintains intracellular potassium homeostasis, supporting membrane potential, pH regulation, and growth, which are essential functions for biofilm development and maintenance [38].

Cytochrome c family proteins are essential for electron transfer, redox balancing, and energy production in bacteria, processes critical for sustaining biofilm metabolic activity [39].

These results align with the phenotypic observations and highlight key biological processes that may underlie variation in biofilm development among isolates. Together, these functional attributes suggest roles for these proteins in supporting *Campylobacter* biofilm matrix production, structural integrity, and stress resilience. Future studies should focus on examining biofilm formation under diverse environmental conditions and across multiple time points, experimentally validating the candidate genes and proteins identified here, characterizing the regulatory networks that govern their activity, and exploring targeted biofilm disruption strategies to extend these findings and help mitigate *Campylobacter* persistence in food production environments. Furthermore, given the close association between biofilm formation and enhanced antimicrobial tolerance, additional research is needed to assess how these mechanisms contribute to antimicrobial resistance and their broader implications for public health.

## 3. Materials and Methods

### 3.1. Sample Preparation

*C. jejuni* and *C. coli* strains were isolated from retail meat, including chicken meat, chicken livers, and beef livers, collected between 2011 and 2023 using previously described methods [40]. Briefly, 450 g of meat was combined with 250 mL buffered peptone water (BPW) and homogenized using a stomacher. The homogenate was centrifuged and the pellet was enriched in Bolton broth supplemented with horse blood and selective antibiotics (cefoperazone, trimethoprim, vancomycin, and cycloheximide) at 42 °C for 24 h under microaerobic conditions (5% O_2_, 10% CO_2_, and 85% N_2_) using a CampyPak (Becton, Dickinson and Company, Franklin Lakes, NJ, USA) in an airtight jar. Following enrichment, passive filtration onto Brucella agar was employed for strain isolation based on the highly motile nature of *Campylobacter*. Colonies were re-streaked twice for strain purification, and genus and species identification was performed using a multiplex qPCR assay previously developed for differentiating *C. jejuni* and *C. coli* [41].

### 3.2. Biofilm Formation

*Campylobacter* isolates were streaked from −80 °C frozen stocks onto Mueller–Hinton (MH) agar plates and incubated overnight under microaerobic conditions (5% O_2_, 10% CO_2_, 85% N_2_) at 42 °C. Fresh colonies were scraped from the agar plates and resuspended in 2 mL of MH broth, followed by overnight incubation under the same conditions. Subsequently, 50 μL of the overnight culture was inoculated into 5 mL of MH broth and homogenized. Two aliquots of 2 mL of the diluted culture were transferred into 10 cm^2^/10 mL polystyrene tissue culture tubes with a flat surface and vent cap (Techno Plastic Products AG, Trasadingen, Canton Schaffhausen, Switzerland) and incubated horizontally for 5 days to allow biofilm development. A 5-day static incubation period was selected for the biofilm development due to the growth variation observed among the *Campylobacter* isolates used in our study. This extended incubation ensures that both fast- and slow-growing strains are able to reach stable biofilm development, thereby allowing consistent and comparable measurements across isolates [15,42]. All incubations were performed under microaerobic conditions at 42 °C.

### 3.3. Biofilm Quantification

Biofilm cultures were filtered onto a 40 μm cell strainer fitted on a 50 mL conical tube. The strainer containing aggregated cells was rinsed with 2 mL of fresh MH broth, transferred to a 6-well plate and then submerged in 6 mL 0.1% crystal violet solution. After a 30 min incubation at room temperature, the strainers and aggregates were rinsed three times with 5 mL of sterile water and photographed in a light box. The strainer was then placed in a fresh 6-well plate and submerged in 6 mL of 100% ethanol. After 30 min of incubation at room temperature with gentle shaking, a pipette was used to dislodge aggregates from the strainer surface and mix the ethanol. The absorbance of the ethanol solution (200 μL in each well, in duplicate) was measured at 590 nm using a Cytation 5 plate reader (BioTek/Agilent Life Sciences, Winooski, VT, USA).

### 3.4. Genome Sequencing, Assembly, and Annotation

Genomic DNA was extracted using the Qiagen genomic tip 100/G kit (Qiagen, Valencia, CA, USA) and quantified with a Qubit 3.0 fluorometer (Thermo Fisher Scientific, Waltham, MA, USA) following the manufacturers’ instructions. Whole-genome sequencing was performed using Illumina MiSeq and Pacific Biosciences (PacBio) RSII and/or Sequel platforms (Illumina, San Diego, CA, USA; Pacific Biosciences, Menlo Park, CA, USA). Genome assemblies were generated from PacBio long reads using Canu v2.2 [43]. Overhangs of assembled contigs were trimmed and reoriented using Circlator v1.5.5 [44] to produce complete circularized genomes.

In a few cases where reorientation and trimming failed, Illumina MiSeq reads were used to correct sequencing errors in assembled contigs: MiSeq reads were mapped to Canu assemblies using BWA v0.7.17-r1188 [45]; errors were corrected using Pilon v1.22 [46]; and Pilon correction was repeated iteratively until no errors were detected. Finally, the assemblies were trimmed and reoriented using Circlator v1.5.5 to generate complete circular genomes.

Table 2 summarizes the source and assembled genome information for *C. jejuni* and *C. coli* isolates. For each strain, the complete genome of approximately 1.6–1.8 Mbp, consistent with the size of previously reported *Campylobacter* chromosomes, was annotated using the RAST server [47,48]. Chromosomal integrity was verified by confirming the presence of the start gene *dnaA*, three copies of rRNA operons (23S, 16S, and 5S rRNA), and minimal repeat sequences. Smaller contigs were assessed for potential plasmid sequences.

### 3.5. Pangenome and Phylogenetic Analysis

The *Campylobacter* pangenome was constructed using the KBase web server [53]. First, genome assemblies were annotated and categorized into the functional subsystems using RASTtk v1.073, then the pangenome was constructed using Ortho-MCL v0.0.8 [54]. To investigate the relatedness of different genome clusters, we constructed a phylogenetic parsimony tree from all SNPs using kSNP4 v4.1 [55] with a k-mer size of 19. Bootstrap support values were calculated with IQ-Tree v 2.1.2 [56] on the CIPRES Scientific Gateway [57]. Briefly, a maximum likelihood consensus tree was built using a General Time Reversible (GTR) model with a correction for ascertainment bias. The kSNP4 parsimony tree served as the starting tree, and a non-parametric bootstrap analysis was performed with 1000 replicates to assess branch support. Complete genome sequences (accession numbers) used for this analysis are listed in Table 2. The resulting phylogenetic tree was visualized using Iroki [58].

To determine which genes were unique or shared among the *Campylobacter* genomes, we constructed a pangenome using OrthoMCL v.2.0, based on RASTtk annotations generated through the KBase Server. Heatmaps depicting gene presence/absence and the number of shared genes among the genomes were generated in R v4.4.0 (R Core Team 2024) using the ggplot2 and viridis packages [59].

## 4. Conclusions

This study demonstrates the strain-specific nature of biofilm formation in *Campylobacter* spp., identifying two strong biofilm-forming strains among 18 food isolates. By integrating whole-genome sequencing with comparative genomic analysis of biofilm-forming and non-forming strains, we uncovered key genes and pathways associated with strong biofilm formation, providing new insight into the potential genetic determinants underlying this phenotype. Phylogenetic and pangenomic analyses provided valuable insights into the genetic basis of biofilm formation and highlighted the strain-specific ability to utilize this mechanism for survival in challenging conditions, particularly those encountered during food processing and storage. Importantly, the identification of proteins linked to both biofilm formation and pathogenesis in *C. jejuni* strains YH001 and YH027 offers promising molecular targets for therapeutic approaches and intervention strategies aimed at disrupting biofilms. Collectively, these findings enhance our understanding of *Campylobacter* persistence in food environments and highlight molecular targets that may inform the development of innovative intervention strategies to reduce contamination and improve food safety.

## Figures and Tables

**Table 1:** Annotated proteins uniquely present in *C. jejuni* strains YH001 and YH027.

Annotated Protein	Subsystem Category	YH001	YH027	Other Strains
Putative Dihydrolipoamide dehydrogenase (EC 1.8.1.4); Mercuric ion reductase (EC 1.16.1.1); PF00070 family, FAD-dependent NAD(P)-disulphide oxidoreductase	Virulence, Disease and Defense	1	1	0
D-alanyl-D-alanine carboxypeptidase (EC 3.4.16.4)	Protein Metabolism	1	1	0
FIG00471123: hypothetical protein	None	1	1	0
DNA-binding protein Roi	None	1	1	0
FIG00470265: hypothetical protein	None	1	1	0
FIG00471635: hypothetical protein	None	1	1	0
FIG00470314: hypothetical protein	None	1	1	0
Hydrogenase, (NiFe)/(NiFeSe) small subunit family	None	1	1	0
L-Proline/Glycine betaine transporter ProP	Amino Acids and Derivatives	1	1	0
Uncharacterized membrane protein, YraQ family	None	1	1	0
Alpha-ketoglutarate permease	None	1	1	0
Putative efflux protein	None	1	1	0
Ammonium transporter	Nitrogen Metabolism	1	1	0
C4-dicarboxylate transporter	None	1	1	0
Sodium-dependent phosphate transporter	Membrane Transport	1	1	0
Multi antimicrobial extrusion protein (Na(+)/drug antiporter), MATE family of MDR efflux pumps	Virulence, Disease and Defense	1	1	0
Hypothetical protein Cj0566	None	1	1	0
Potassium-transporting ATPase A chain (EC 3.6.3.12) (TC 3.A.3.7.1)	Potassium Metabolism	1	1	0
Cytochrome c family protein	None	1	1	0

Protein names and subsystem categories were assigned based on annotation using the RAST server. A designation of ‘None’ in the Subsystem Category indicates that the protein’s functional subsystem could not be identified by the RAST annotation. The values “1” and “0” indicated the presence and absence of the genes in the isolates, respectively.

**Table 2:** Sources and genome information of *C. jejuni* and *C. coli* isolates.

Strain and Species	Source	Genome Size (bp)	%GC	Accession No.	Reference
*C. jejuni* YH001	Veal livers	1,712,361	30.5	CP010058	[49]
*C. jejuni* YH008	Drumsticks	1,792,424	30.5	CP172380	This work
*C. jejuni* (S27Cj) YH009	Chicken thighs	1,663,226	30.5	CP131444	[50]
*C. jejuni* (S33Cj) YH010	Chicken thighs	1,748,761	30.5	CP131442	[50]
*C. jejuni* YH012	Chicken livers	1,698,963	30.5	CP172815	This work
*C. jejuni* YH013	Chicken livers	1,691,848	30.5	CP172379	This work
*C. jejuni* YH014	Chicken livers	1,802,039	30.5	CP172376	This work
*C. jejuni* YH027	Calf livers	1,710,959	30.5	CP172352	This work
*C. jejuni* YH028	Beef livers	1,667,698	30.5	CP172351	This work
*C. coli* YH501	Drumsticks	1,668,523	31.5	CP015528	[51]
*C. coli* YH502	Drumsticks	1,718,974	31.0	CP018900	[52]
*C. coli* YH504	Drumsticks	1,722,143	31.0	CP091644	[51]
*C. coli* YH507	Chicken livers	1,756,096	31.0	CP172392	This work
*C. coli* YH508	Chicken thighs	1,703,740	31.5	CP172391	This work
*C. coli* YH509	Chicken livers	1,697,113	31.5	CP172390	This work
*C. coli* YH510	Chicken livers	1,812,356	31.0	CP172387	This work
*C. coli* YH511	Chicken livers	1,674,288	31.5	CP172385	This work
*C. coli* YH512	Chicken livers	1,754,135	31.5	CP172384	This work

## Data Availability

All the assembled genome sequences of *Campylobacter* isolates were deposited and are available in GenBank, NCBI under the accession numbers listed in Table 2.

## Supplementary Materials

The following supporting information can be downloaded at: https://www.mdpi.com/article/10.3390/ijms27062543/s1.

## References

[1] Centers for Disease Control and Prevention About *Campylobacter* Infection. https://www.cdc.gov/campylobacter/about/index.html.

[2] Tikhomirova A., McNabb E.R., Petterlin L., Bellamy G.L., Lin K.H., Santoso C.A., Daye E.S., Alhaddad F.M., Lee K.P., Roujeinikova A. (2024). *Campylobacter jejuni* virulence factors: Update on emerging issues and trends. J. Biomed. Sci..

[3] World Health Organization (WHO) Fact Sheet on *Campylobacter*. https://www.who.int/news-room/fact-sheets/detail/campylobacter.

[4] Veronese P., Dodi I. (2024). *Campylobacter jejuni*/*coli* Infection: Is It Still a Concern?. Microorganisms.

[5] Karygianni L., Ren Z., Koo H., Thurnheer T. (2020). Biofilm Matrixome: Extracellular Components in Structured Microbial Communities. Trends Microbiol..

[6] Ryder C., Byrd M., Wozniak D.J. (2007). Role of polysaccharides in *Pseudomonas aeruginosa* biofilm development. Curr. Opin. Microbiol..

[7] Lasa I., Penades J.R. (2006). Bap: A family of surface proteins involved in biofilm formation. Res. Microbiol..

[8] Fong J.N.C., Yildiz F.H. (2015). Biofilm Matrix Proteins. Microbiol. Spectr..

[9] Panlilio H., Rice C.V. (2021). The role of extracellular DNA in the formation, architecture, stability, and treatment of bacterial biofilms. Biotechnol. Bioeng..

[10] Bai X., Nakatsu C.H., Bhunia A.K. (2021). Bacterial Biofilms and Their Implications in Pathogenesis and Food Safety. Foods.

[11] Olsen I. (2015). Biofilm-specific antibiotic tolerance and resistance. Eur. J. Clin. Microbiol. Infect. Dis..

[12] Buswell C.M., Herlihy Y.M., Lawrence L.M., McGuiggan J.T., Marsh P.D., Keevil C.W., Leach S.A. (1998). Extended survival and persistence of *Campylobacter* spp. in water and aquatic biofilms and their detection by immunofluorescent-antibody and -rRNA staining. Appl. Environ. Microbiol..

[13] Teh A.H., Lee S.M., Dykes G.A. (2014). Does *Campylobacter jejuni* form biofilms in food-related environments?. Appl. Environ. Microbiol..

[14] Reeser R.J., Medler R.T., Billington S.J., Jost B.H., Joens L.A. (2007). Characterization of *Campylobacter jejuni* biofilms under defined growth conditions. Appl. Environ. Microbiol..

[15] Ica T., Caner V., Istanbullu O., Nguyen H.D., Ahmed B., Call D.R., Beyenal H. (2012). Characterization of mono- and mixed-culture *Campylobacter jejuni* biofilms. Appl. Environ. Microbiol..

[16] Melo R.T., Mendonca E.P., Monteiro G.P., Siqueira M.C., Pereira C.B., Peres P., Fernandez H., Rossi D.A. (2017). Intrinsic and Extrinsic Aspects on *Campylobacter jejuni* Biofilms. Front. Microbiol..

[17] Silha D., Sirotkova S., Svarcova K., Hofmeisterova L., Korycanova K., Silhova L. (2021). Biofilm Formation Ability of Arcobacter-like and *Campylobacter* Strains under Different Conditions and on Food Processing Materials. Microorganisms.

[18] Puning C., Su Y., Lu X., Golz G. (2021). Molecular Mechanisms of *Campylobacter* Biofilm Formation and Quorum Sensing. Curr. Top. Microbiol. Immunol..

[19] Korkus J., Salata P., Thompson S.A., Paluch E., Bania J., Walecka-Zacharska E. (2024). The role of cydB gene in the biofilm formation by *Campylobacter jejuni*. Sci. Rep..

[20] Svensson S.L., Pryjma M., Gaynor E.C. (2014). Flagella-mediated adhesion and extracellular DNA release contribute to biofilm formation and stress tolerance of *Campylobacter jejuni*. PLoS ONE.

[21] Sulaeman S., Le Bihan G., Rossero A., Federighi M., De E., Tresse O. (2010). Comparison between the biofilm initiation of *Campylobacter jejuni* and *Campylobacter coli* strains to an inert surface using BioFilm Ring Test. J. Appl. Microbiol..

[22] Sauer K., Camper A.K., Ehrlich G.D., Costerton J.W., Davies D.G. (2002). *Pseudomonas aeruginosa* displays multiple phenotypes during development as a biofilm. J. Bacteriol..

[23] Flemming H.C., van Hullebusch E.D., Little B.J., Neu T.R., Nielsen P.H., Seviour T., Stoodley P., Wingender J., Wuertz S. (2025). Microbial extracellular polymeric substances in the environment, technology and medicine. Nat. Rev. Microbiol..

[24] Selles Vidal L., Kelly C.L., Mordaka P.M., Heap J.T. (2018). Review of NAD(P)H-dependent oxidoreductases: Properties, engineering and application. Biochim. Biophys. Acta Proteins Proteom..

[25] Pal S., Jain D., Biswal S., Rastogi S.K., Kumar G., Ghosh A.S. (2024). The physiological role of *Acinetobacter baumannii* DacC is exerted through influencing cell shape, biofilm formation, the fitness of survival, and manifesting DD-carboxypeptidase and beta-lactamase dual-enzyme activities. FEMS Microbiol. Lett..

[26] Peters K., Kannan S., Rao V.A., Biboy J., Vollmer D., Erickson S.W., Lewis R.J., Young K.D., Vollmer W. (2016). The Redundancy of Peptidoglycan Carboxypeptidases Ensures Robust Cell Shape Maintenance in *Escherichia coli*. mBio.

[27] Iwata T., Watanabe A., Kusumoto M., Akiba M. (2016). Peptidoglycan Acetylation of *Campylobacter jejuni* Is Essential for Maintaining Cell Wall Integrity and Colonization in Chicken Intestines. Appl. Environ. Microbiol..

[28] Das T., Sehar S., Manefield M. (2013). The roles of extracellular DNA in the structural integrity of extracellular polymeric substance and bacterial biofilm development. Environ. Microbiol. Rep..

[29] Greening C., Biswas A., Carere C.R., Jackson C.J., Taylor M.C., Stott M.B., Cook G.M., Morales S.E. (2016). Genomic and metagenomic surveys of hydrogenase distribution indicate H2 is a widely utilised energy source for microbial growth and survival. ISME J..

[30] Kapfhammer D., Karatan E., Pflughoeft K.J., Watnick P.I. (2005). Role for glycine betaine transport in *Vibrio cholerae* osmoadaptation and biofilm formation within microbial communities. Appl. Environ. Microbiol..

[31] Typas A., Banzhaf M., Gross C.A., Vollmer W. (2011). From the regulation of peptidoglycan synthesis to bacterial growth and morphology. Nat. Rev. Microbiol..

[32] Doucette C.D., Schwab D.J., Wingreen N.S., Rabinowitz J.D. (2011). α-Ketoglutarate coordinates carbon and nitrogen utilization via enzyme I inhibition. Nat. Chem. Biol..

[33] Piddock L.J. (2006). Multidrug-resistance efflux pumps—Not just for resistance. Nat. Rev. Microbiol..

[34] Zhang L., Mah T.F. (2008). Involvement of a novel efflux system in biofilm-specific resistance to antibiotics. J. Bacteriol..

[35] Ardin A.C., Fujita K., Nagayama K., Takashima Y., Nomura R., Nakano K., Ooshima T., Matsumoto-Nakano M. (2014). Identification and functional analysis of an ammonium transporter in Streptococcus mutans. PLoS ONE.

[36] Neznansky A., Blus-Kadosh I., Yerushalmi G., Banin E., Opatowsky Y. (2014). The *Pseudomonas aeruginosa* phosphate transport protein PstS plays a phosphate-independent role in biofilm formation. FASEB J..

[37] Alav I., Sutton J.M., Rahman K.M. (2018). Role of bacterial efflux pumps in biofilm formation. J. Antimicrob. Chemother..

[38] Epstein W. (2003). The roles and regulation of potassium in bacteria. Prog. Nucleic Acid. Res. Mol. Biol..

[39] Gray H.B., Winkler J.R. (2010). Electron flow through metalloproteins. Biochim. Biophys. Acta.

[40] He Y., Capobianco J., Armstrong C.M., Chen C.Y., Counihan K., Lee J., Reed S., Tilman S. (2024). Detection and Isolation of *Campylobacter* spp. from Raw Meat. J. Vis. Exp..

[41] He Y.P., Yao X.M., Gunther N.W., Xie Y.P., Tu S.I., Shi X.M. (2010). Simultaneous Detection and Differentiation of *Campylobacter jejuni*, C. coli, and C. lari in Chickens Using a Multiplex Real-Time PCR Assay. Food Anal. Method..

[42] Gunther N.W., Nunez A., Bagi L., Abdul-Wakeel A., Ream A., Liu Y., Uhlich G. (2023). Butyrate decreases *Campylobacter jejuni* motility and biofilm partially through influence on LysR expression. Food Microbiol..

[43] Koren S., Walenz B.P., Berlin K., Miller J.R., Bergman N.H., Phillippy A.M. (2017). Canu: Scalable and accurate long-read assembly via adaptive k-mer weighting and repeat separation. Genome Res..

[44] Hunt M., Silva N.D., Otto T.D., Parkhill J., Keane J.A., Harris S.R. (2015). Circlator: Automated circularization of genome assemblies using long sequencing reads. Genome Biol..

[45] Li H., Durbin R. (2009). Fast and accurate short read alignment with Burrows-Wheeler transform. Bioinformatics.

[46] Walker B.J., Abeel T., Shea T., Priest M., Abouelliel A., Sakthikumar S., Cuomo C.A., Zeng Q., Wortman J., Young S.K. (2014). Pilon: An integrated tool for comprehensive microbial variant detection and genome assembly improvement. PLoS ONE.

[47] Aziz R.K., Bartels D., Best A.A., DeJongh M., Disz T., Edwards R.A., Formsma K., Gerdes S., Glass E.M., Kubal M. (2008). The RAST Server: Rapid annotations using subsystems technology. BMC Genom..

[48] Overbeek R., Olson R., Pusch G.D., Olsen G.J., Davis J.J., Disz T., Edwards R.A., Gerdes S., Parrello B., Shukla M. (2014). The SEED and the Rapid Annotation of microbial genomes using Subsystems Technology (RAST). Nucleic Acids Res..

[49] He Y., Yan X., Reed S., Xie Y., Chen C.Y., Irwin P. (2015). Complete Genome Sequence of *Campylobacter jejuni* YH001 from Beef Liver, Which Contains a Novel Plasmid. Genome Announc..

[50] He Y., Kanrar S., Reed S., Lee J., Capobianco J. (2024). Whole Genome Sequences, De Novo Assembly, and Annotation of Antibiotic Resistant *Campylobacter jejuni* Strains S27, S33, and S36 Newly Isolated from Chicken Meat. Microorganisms.

[51] He Y., Reed S., Yan X., Zhang D., Strobaugh T., Capobianco J., Gehring A. (2022). Complete Genome Sequences of Multidrug-Resistant *Campylobacter coli* Strains YH501, YH503, and YH504, from Retail Chicken. Microbiol. Resour. Announc..

[52] Ghatak S., He Y., Reed S., Strobaugh T., Irwin P. (2017). Whole genome sequencing and analysis of *Campylobacter coli* YH502 from retail chicken reveals a plasmid-borne type VI secretion system. Genom. Data.

[53] Arkin A.P., Cottingham R.W., Henry C.S., Harris N.L., Stevens R.L., Maslov S., Dehal P., Ware D., Perez F., Canon S. (2018). KBase: The United States Department of Energy Systems Biology Knowledgebase. Nat. Biotechnol..

[54] Li L., Stoeckert C.J., Roos D.S. (2003). OrthoMCL: Identification of ortholog groups for eukaryotic genomes. Genome Res..

[55] Hall B.G., Nisbet J. (2023). Building Phylogenetic Trees from Genome Sequences With kSNP4. Mol. Biol. Evol..

[56] Nguyen L.T., Schmidt H.A., von Haeseler A., Minh B.Q. (2015). IQ-TREE: A fast and effective stochastic algorithm for estimating maximum-likelihood phylogenies. Mol. Biol. Evol..

[57] Miller M.A., Pfeiffer W., Schwartz T. (2010). Creating the CIPRES Science Gateway for inference of large phylogenetic trees. Proceedings of the Gateway Computing Environments Workshop (GCE), New Orleans, LA, USA, 14 November 2010.

[58] Moore R.M., Harrison A.O., McAllister S.M., Polson S.W., Wommack K.E. (2020). Iroki: Automatic customization and visualization of phylogenetic trees. PeerJ.

[59] Ginestet C. (2011). ggplot2: Elegant Graphics for Data Analysis. J. R. Stat. Soc. A.

